# Developing consensus treatment plans for proliferative lupus nephritis in childhood-onset systemic lupus erythematous

**DOI:** 10.1186/1546-0096-10-S1-A31

**Published:** 2012-07-13

**Authors:** Rina Mina, Hermine Brunner, Barbara Anne Eberhard, Marilynn G Punaro, Stacy P Ardoin, Marisa S Klein-Gitelman, Linda Wagner-Weiner, Lakshmi N Moorthy, Joyce J Hsu, Eyal Muscal, Suhas M Radhakrishna, Laura E Schanberg, Carol A Wallace, Norman T Ilowite, Emily Von Scheven

**Affiliations:** 1 Baylor College of Medicine, Houston, TX, USA; 2 Children's Hospital Montefiore, Bronx, NY, USA; 3 Childrens Memorial Hospital/NW University, Chicago, IL, USA; 4 Cincinnati Child Hospital Medical Center, Cincinnati, OH, USA; 5 Cincinnati Children's Med Ctr, Cincinnati, OH, USA; 6 Cohen Children's Hospital Medical Center, New York, NY, USA; 7 Duke University Medical Center, Durham, NC, USA; 8 Metuchen, NJ, USA; 9 Los Angeles, CA, USA; 10 Dallas, TX, USA; 11 Chicago, IL, USA; 12 Ohio State University, Columbus, OH, USA; 13 Seattle Children's Hospital & Regional Medicine, Seattle, WA, USA; 14 Stanford University, Palo Alto, CA, USA; 15 University of CA San Francisco, San Francisco, CA, USA

## Purpose

The SLE Subcommittee of the Childhood Arthritis and Rheumatology Research Alliance (CARRA) is developing standardized treatment plans for proliferative lupus nephritis (LN) in childhood-onset SLE (cSLE) which will serve as the basis for future comparative effectiveness studies. The Initial Delphi survey revealed wide variability in the treatment of LN in cSLE. This abstract decribes the process of developing standardized evidence-based induction treatment plans for LN in cSLE by using consensus methods.

## Methods

A consensus conference attended by 12 trainees and 42 voting members of the CARRA SLE Subcommittee was conducted to discuss the components of the LN treatment plan for which there was wide variability and poor agreement. After the face-to-face conference, a second online survey focusing on management aspects of the induction therapy for LN was sent to the 42 voting members of the SLE Subcommittee of CARRA to resolve remaining issues. Consensus was defined at 70%.

## Results

At the conference, two immunosuppressive treatment options for the 6-month induction phase, cyclophosphamide and mycophenolate mofetil, were selected. Three steroid regimens (primarily intravenous, primarily oral, or mixed intravenous/oral) with corresponding tapering schedules were developed to reduce variability in steroid exposure *[see Figure A]* Consensus was attained on: a) inclusion and exclusion criteria; b) primary and secondary outcome measures; and c) time-points for assessing patient response. No consensus was reached on the following points: a) age cut-off for the definition of childhood-onset SLE; b) need for SLE-specific quality of life measure; and c) measures of adherence to be utilized. These are being addressed through subsequent surveys.

## Conclusion

Several important consensus points were achieved in the development of induction treatment plans for proliferative lupus nephritis in childhood-onset SLE. Further refinement of these treatment plans and development of plans for maintenance therapy are needed to allow their use in future studies aimed at optimizing therapy for lupus nephritis.

## Disclosure

Rina Mina: NIH, 2; Hermine Brunner: NIAMS-NIH, 2; Barbara Anne Eberhard: None; Marilynn G. Punaro: None; Stacy P. Ardoin: None; Marisa S. Klein-Gitelman: None; Linda Wagner-Weiner: None; Lakshmi N. Moorthy: None; Joyce J. Hsu: None; Eyal Muscal: None; Suhas M. Radhakrishna: None; Laura E. Schanberg: Pfizer Inc, 2; Carol A. Wallace: None; Norman T. Ilowite: None; Emily Von Scheven: None.

